# Epigenetic reprogramming by naïve conditions establishes an irreversible state of partial X chromosome reactivation in female stem cells

**DOI:** 10.18632/oncotarget.25353

**Published:** 2018-05-18

**Authors:** Alexandra V. Panova, Alexandra N. Bogomazova, Maria A. Lagarkova, Sergey L. Kiselev

**Affiliations:** ^1^ Vavilov Institute of General Genetics, Russian Academy of Sciences, Moscow 119991, Russia; ^2^ Scientific-Research Institute of Physical-Chemical Medicine, Moscow 119435, Russia

**Keywords:** X chromosome inactivation, naïve, reprogramming, pluripotency, 5-hydroxymethylcytosine

## Abstract

Female human pluripotent stem cells (PSCs) have variable X-chromosome inactivation (XCI) status. One of the X chromosomes may either be inactive (Xi) or display some active state markers. Long-term cultivation of PSCs may lead to an erosion of XCI and partial X reactivation. Such heterogeneity and instability of XCI status might hamper the application of human female PSCs for therapy or disease modeling. We attempted to address XCI heterogeneity by reprogramming human embryonic stem cells (hESCs) to the naïve state. We propagated five hESC lines under naïve culture conditions. PSCs acquired naïve cells characteristics although these changes were not uniform for all of the hESC lines. Transition to the naïve state was accompanied by a loss of *XIST* expression, loss of Xi H3K27me3 enrichment and a switch in Xi replication synchronously with active X, except for two regions. This pattern of Xi reactivation was observed in all cells in two hESC lines. However, these cells were unable to undergo classical XCI upon spontaneous differentiation. We conclude that naïve culture conditions do not resolve the variability in XCI status in female human ESC lines and establish an irreversible heterogeneous pattern of partial X reactivation.

## INTRODUCTION

X chromosome inactivation (XCI) occurs during early embryonic development in mammalian females. This process equalizes the dose of X-linked genes between males and females. The inactive state of the X chromosome is preserved throughout cell division in female somatic cells. Mouse preimplantation epiblast cells and mouse embryonic stem cells (mESCs) possess two transcriptionally active X chromosomes, and one of these two X chromosomes is inactivated after implantation [1]. Cell lines derived from mouse postimplantation epiblasts (EpiSCs) have one inactivated X chromosome, and they are bFGF-dependent in contrast to LIF-dependent mESCs. EpiSCs are Oct4-positive and have epithelial morphology, reduced Rex1 expression, and retained pluripotency, i.e., they give rise to all three germ layers [2, 3]. These two distinct states of pluripotency are known as “naïve” and “primed” pluripotent states [4]. Human ESCs (hESCs) share many molecular and morphological characteristics with mouse EpiSCs and thus have been considered to exist in the “primed” pluripotent state. Most human pluripotent stem cell (hPSC) lines carry inactive X (Xi) chromosomes, although some cell lines exhibit active state markers on both X chromosomes [5–7]. Culture conditions may lead to partial reactivation of the Xi in hPSCs. This so-called “erosion” process appears to be a frequent event of epigenetic instability in culture and is characterized by the loss of common XCI markers and up-regulation of a subset of X-linked genes [8–10].

Mouse EpiSCs can be converted to a naïve state by KLF4 overexpression and cultivation in the presence of LIF and 2i culture conditions in which the medium is supplemented with small molecules (PD184352 and CHIR99021) that inhibit the MEK and GSK3 signaling pathways. This conversion is accompanied by Xi reactivation [11]. Moreover, it has been reported that X reactivation from mouse EpiSCs can be accomplished when EpiSCs are cultured on feeder cells in the presence of a serum supplement [12]. The resemblance of mouse EpiSCs to hESCs has raised the question as to whether hPSCs may be captured in a “naïve” pluripotent state. Adding 2i and LIF to hESCs and overexpressing the Oct4, SOX2, Klf4, and Klf2 transcription factors has been suggested to convert these cells from primed to an mESC-like naïve state [13]. Several groups have proposed transgene-independent strategies for transitioning hESCs to a naïve pluripotent state [14–19]. The approach used in these studies was based on screening combinations of small molecules and growth factors, although the inhibition of MEK and GSK (2i) was an indispensable feature of these culture conditions. Searches for optimal naïve culture conditions have been based on either morphological mESC-like features [16, 17] or reporter systems used to maintain the Oct4 distal enhancer [18], express Oct4-GFP [15], or enhance NANOG expression [14]. One group captured naïve hESCs by dissociating the inner cell mass (ICM) directly into single cells to separate epiblasts from the primitive endoderm [20]. Comparisons of captured naïve hPSCs with mESCs or human blastocysts using transcriptome data [15, 18], methylation profiling [15, 20], metabolic properties and morphology have demonstrated the naïve characteristics of these cell lines. However, in contrast to mESCs, no verified X chromosome reactivation has been observed in naïve hPSCs in these studies. Of note, there is no uniform criterion or common approach to characterizing XCI status. The presence of H3K27me3foci in the nucleus is the most common marker of Xi but does not always reflect the XCI status [8–10]. In most studies, the generation of naïve hPSC reactivation was evaluated by indirect markers such as a decrease in the percentage of cells with H3K27me3 foci or the presence of *XIST* clouds or methylation of the *XIST* promoter [16, 17, 19, 21]. Although histone marks and *XIST* expression indicate changes ongoing during XCI, they do not provide evidence for functional changes to the epigenetic state.

The only study that verified reactivation of a previously inactive X chromosome in 5iLAF naïve conditions was performed on UCLA1 cell line using single-cell RNA-seq and RNA-FISH. However, the reactivation was accompanied by chromosome-wide transcriptional dampening [22]. Later, 5iL/A/F18 and 2i/L+PKCi19 culture conditions were used to investigate the X inactivation state in two female ESC lines (UCLA1 and transgenic H9) where biallelic expression of X-linked genes was observed [22–24]. Another comprehensive study of X reactivation upon transition to the naïve state was performed using Naïve Human Stem cell Medium (NHSM, containing PD0325901, CHIR99021, SP600125, SB203580) conditions [15]. The authors observed the disappearance of H3K27me3 foci and *XIST* clouds, as well as changes to *XIST* gene methylation patterns in naïve PSCs. Most importantly, they showed that human cells acquire H3K27me3 foci upon differentiation from a naïve PSC state. NHSM-hESCs acquire the capacity to contribute to human-mouse chimeras [15], and NHSM-hPSCs robustly engraft in both pig and cattle pre-implantation blastocysts [25]. The ability to integrate into the ICM of a blastocyst is one of the most reliable indicators of the ICM-like state of NHSM-maintained cells; however, the XCI status under NHSM conditions remains largely unclear.

Another functional characteristic of Xi that has barely been investigated during reprogramming is replication. The Xi replicates during the S phase later, which is later than the active chromosome [26, 27]. Recently, we demonstrated that during reprogramming, the Xi can switch from late to synchronous replication with restoration of the transcription of previously silenced genes. This process is accompanied by the accumulation of a new epigenetic mark of the DNA demethylation pathway, 5-hydroxymethylcytosine (5-hmC), on the reactivated X chromosome [28]. However, there are still some regions that escape the switch to synchronous replication in the reprogrammed cells. X chromosome status has been investigated in depth under 5iL/A/F18 and 2i/L+PKCi19 conditions, and biallelic expression of X-linked genes was observed in some selected cell lines [22–24]. Therefore, we decided to investigate whether epigenetic reprogramming to the naïve state by NHSM medium that have been previously shown efficiently reprogram a number of hPSC lines can uniformly lead to reactivation of Xi in hESCs thereby resetting the problem of XCI erosion [29]. We transferred five previously described female hESC lines from conventional culture conditions to the NHSM medium and evaluated the loss of XCI in NHSM-hESCs by loss of *XIST*, H3K27me3 foci, by replication timing analysis, 5-hmc acquisition, and their ability to undergo XCI upon differentiation.

## RESULTS

### XCI characteristics in primed hESC lines

**Figure 1:**
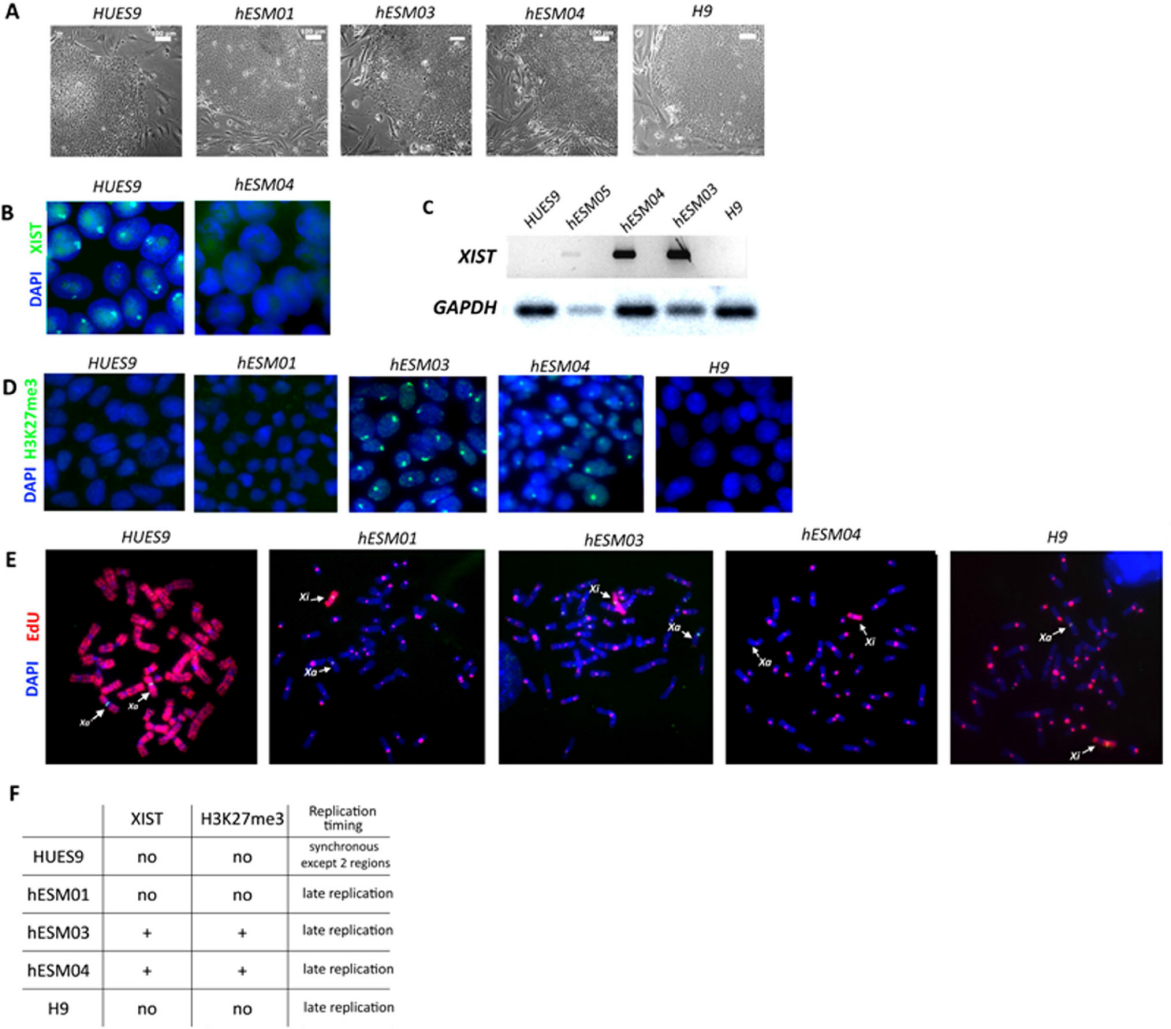
Xi characterization in primed hESC lines (**A**) Flat morphology of the colonies of the primed hESC lines on feeder cells. Phase contrast. Scale bar, 100 μm. (**B**) RNA-FISH analysis of primed hESCs for XIST expression (green). The nuclei are counterstained with DAPI (blue). (**C**) RT-PCR with primers specific for the XIST gene. (**D**) Cell lines were analyzed with H3K27me3 antibodies. The nuclei were counterstained with DAPI (blue). (**E**) Analysis of X-chromosome replication timing in the cell lines by EdU pulse-labeling (red). Synchronously replicating X chromosomes in the HUES9 cell line and late-replicating X chromosome in four other cell lines are indicated by arrows. Metaphase chromosomes are counterstained with DAPI (blue). (**F**) Table summarizing data on XIST expression and H3K27me3 foci and replication timing in 5 primed hESC lines.

In our study, five female hESC lines, namely, hESM01, hESM03, hESM04, H9 and HUES9, were used for the analysis. Cell colonies cultured in 20% KO serum replacement on feeder layers had typical morphology indicative of primed hESCs (Figure 1A). To characterize XCI, we used several epigenetic non-direct marks of inactivation, including *XIST*, which was detected by RNA-FISH. An *XIST*-cloud was detected in the majority of cells in the hESM03 and hESM04 cell lines, and no *XIST* coating was observed in the hESM01, H9 or HUES9 cell lines (Figure 1B, 1C, 1F). Next, we examined the cell lines for histone H3K27me3 marks on Xi using immunocytochemistry. The same two cell lines (hESM03 and hESM04) that demonstrated *XIST* expression exhibited H3K27me3 foci (Figure 1D, 1F). In the hESM01, H9 and HUES9 cell lines, we did not observe any foci indicative of the heterochromatin histone modification H3K27me3 (Figure 1D, 1F).

Xi heterochromatin is replicated during the late S-phase of the cell cycle [30]. Previously, we confirmed that replication timing analysis is a reliable marker of XCI in PSCs [28]. To detect replication timing, we applied pulse labeling with 5-ethynyl-2´-deoxyuridine (EdU) to five primed hESC lines and then analyzed EdU incorporation in the X chromosomes of metaphase cells. We considered replication to be late if EdU incorporation was observed in the pericentromeric regions of autosomes. The inactive X in primed hESM03, hESM01, hESM04 and H9 cells replicated almost entirely during the late S phase (Figure 1E, 1F). These four cell lines were considered to be cell lines with Xi. The HUES9 cell line was considered to have partially active or highly eroded Xi as the Xi in primed HUES9 cells replicated synchronously with the active X except for only two bands (Figure 1E, 1F). Thus, we characterized two cell lines with classical XCI (hESM03, hESM04), two cell lines with Xi with signs of erosion due to the absence of *XIST* and H3K27me3 foci (hESM01, H9), and one cell line with chromosome-wide changes in Xi state (HUES9).

### Characteristics of human ESC lines upon naïve culture conditions

**Figure 2:**
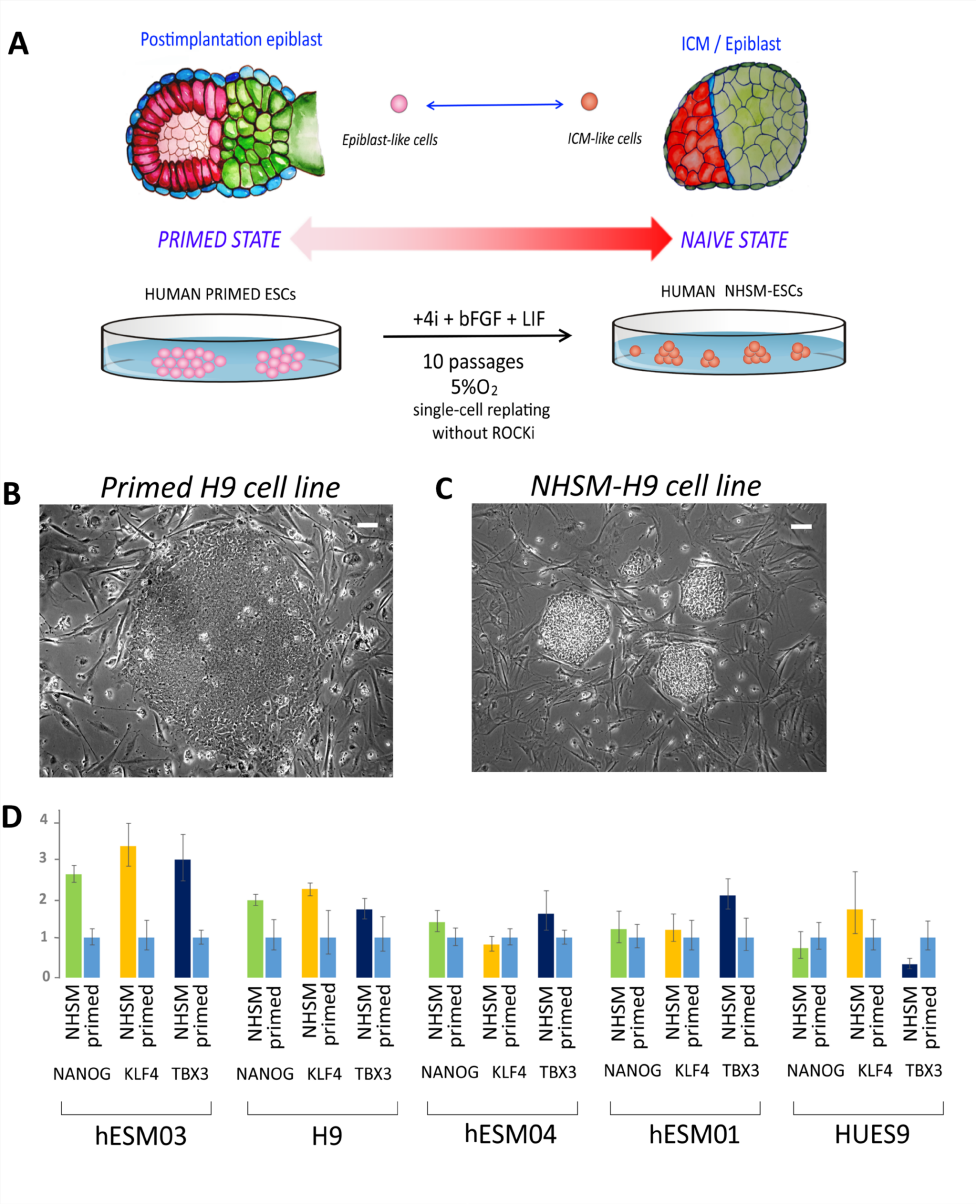
NHSM-hESCs reprogramming and characterization (**A**) Schematic representation of the experimental transition of hESCs from primed to naïve states. ES medium was substituted for NHSM, and the cell lines were maintained for 10 passages before their analysis. (**B**) Flat morphology of the colonies of the primed hESC line on feeder cells (H9 cell line is shown). Phase contrast. Scale bar, 100 μm. (**C**) Dome-shaped morphology of cell colonies after transition to a naïve state (NHSM-H9 cell line after 8 passages is shown). Phase contrast. Scale bar, 100 μm. (**D**) Relative expression of naïve-associated genes *NANOG*, *KLF4*, and *TBX3* shows the transition to a naïve state in hESM03 and H9 cell-lines. Expression level was normalized to *GAPDH* expression level.

To convert primed hESCs to naïve state cells, ESC medium for conventional cultivation (see Materials and Methods) was replaced with the previously described naïve medium (NHSM) [15], and the cells were grown in a 5% O_2_ and 5% CO_2_ humidified atmosphere (Figure 2A). The cell lines were maintained under naïve conditions for 10 passages before analysis.

Morphological changes were first detected at 4–6 days after the transfer of cells to NHSM conditions. Cell colonies acquired tight edges and “domed” shapes (Figure 2B, 2C; Supplementary Figure 1). Additionally, the cell lines were effectively replated by trypsinization for 10 passages without any need for ROCK-inhibitor Y-27632, except in the hESM04 line, for which replating by single-cell dissociation was only successful in the presence of the ROCK inhibitor.

To characterize the degree of transition to the naïve state, we analyzed the expression of genes considered to be upregulated in the naïve state [17, 18]. Real-time PCR data showed that the naïve-associated *NANOG*, *KLF4*, and *TBX3* genes were up-regulated in hESM03 and H9 cells, but not in hESM04, HUES9 or hESM01 cells (Figure 2D), again indicating heterogeneity among the cell lines in the ability to transition to the naïve state.

**Figure 3:**
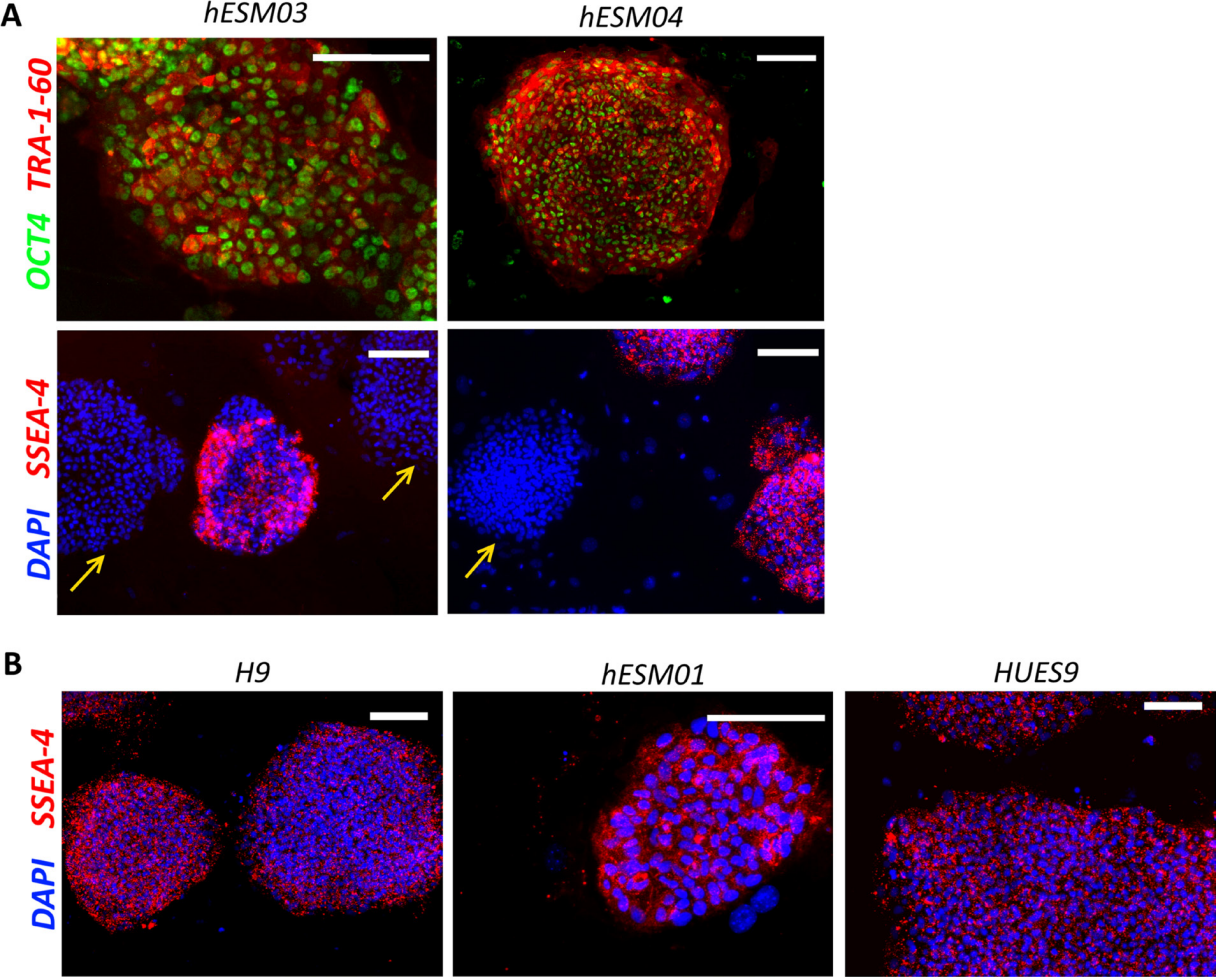
Retention of pluripotency in NHSM-hESCs (**A**) Immunofluorescent staining with OCT4 (green), TRA-1-60 (red) and SSEA-4 (red) antibodies in NHSM-hESM03 and NHSM-hESM04 cell lines. Scale bar, 100 μm. Heterogeneity of SSEA-4 (red) staining in naïve culture conditions in NHSM-hESM03 and NHSM-hESM04 cell lines is shown. Arrows indicate SSEA-4-negative colonies in NHSM conditions. (**B**) NHSM-H9, NHSM-HUES9, NHSM-hESM01 cell lines homogenously stained with SSEA-4. The nuclei are stained with DAPI (blue). Scale bar, 100 μm.

All five ESC lines were Oct4 and TRA-1-60 immunopositive (Figure 3A). After cultivation under NHSM conditions, the hESM03 and hESM04 cell lines showed heterogeneity in SSEA-4 immunostaining (Figure 3A, 3B). These cell lines had both SSEA-4-positive and -negative colonies. Earlier, SSEA-4 staining heterogeneity was not detected upon application of NHSM conditions [15], although later studies demonstrated SSEA-4 heterogeneity in 5i culture conditions [18], and most remarkably, SSEA-4-negative cells were postulated to be truly naïve according to their methylation profile [31].

Thus, cultivation of primed hESCs in naïve conditions led to naïve-like morphological changes in all cell lines. However, only two cell lines (NHSM-hESM03 and NHSM-H9) demonstrated up-regulation of naïve-associated genes and increased single-cell survival characteristics.

### Changes of XCI state in ESCs cultured under NHSM conditions

**Figure 4:**
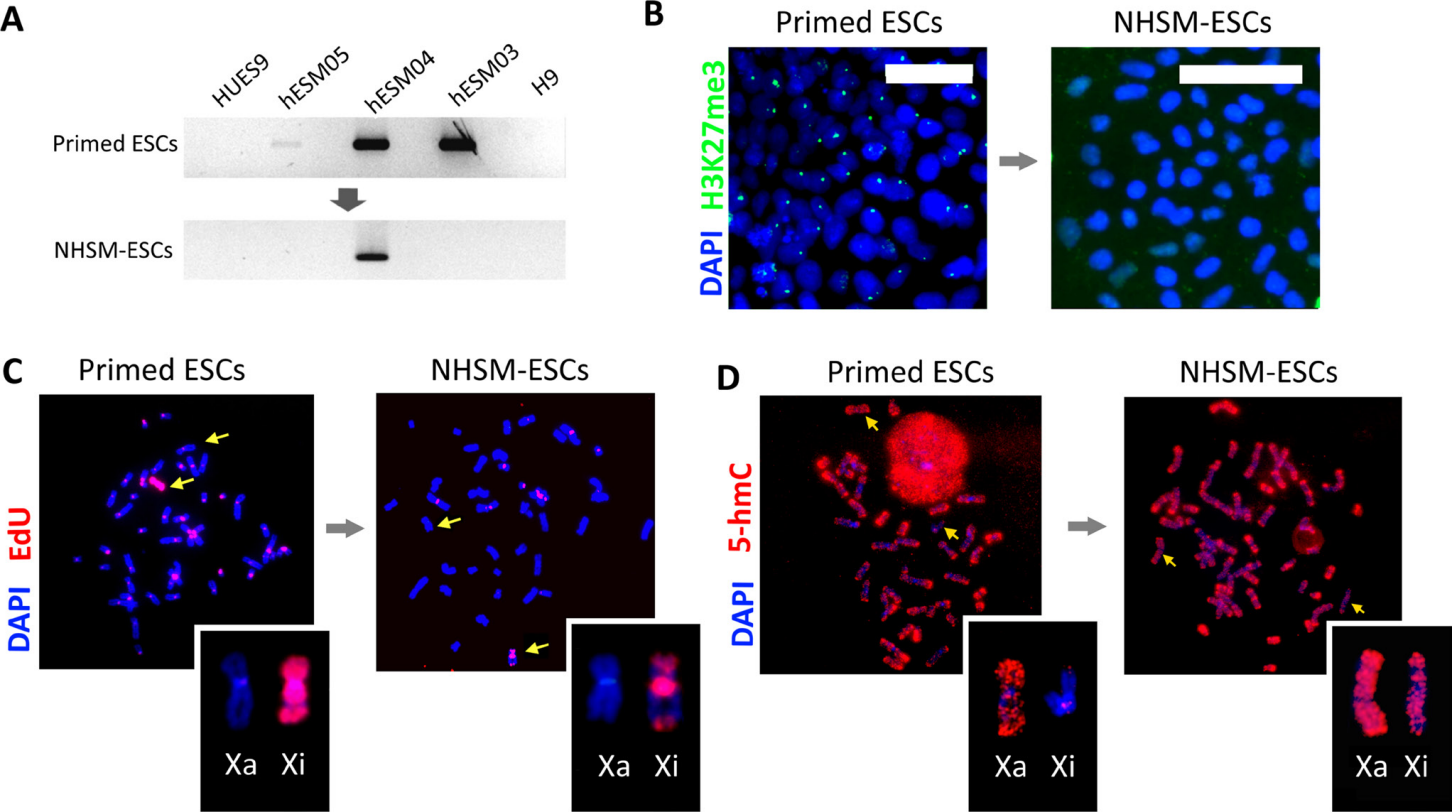
X chromosome inactivation status and replication pattern upon naïve culture conditions (**A**) Reprogramming to naïve state leads to *XIST* silencing. RT-PCR with primers specific for the *XIST* gene demonstrated the loss of *XIST* expression in the NHSM-hESM03 cell line (see full size gels in Supplementary Information). GAPDH was used for RNA normalization (not shown). (**B**) Reprogramming to a naïve state erases the H3K27me3 suppressive histone mark. Immunostaining with H3K27me3 (left, green) reveals positively stained foci in the primed hESM03 cell line that disappear after 10 passages in NHSM (right). The nuclei were stained with DAPI (blue). Scale bar 50 μm. (**C**) Reprogramming to a naïve state leads to a shift in replication timing of the X chromosome from late to early S-phase (the NHSM-hESM03 cell line is shown). Pulse-labeling with EdU (red) demonstrated that after 10 passages in NHSM, two regions of the Xi replicate synchronously with the Xa. The metaphase chromosomes were counterstained with DAPI (blue). (**D**) Reprogramming to naïve state for 10 passages leads to the hypomethylation of Xi. Immunostating with 5-hmC antibodies (red) (NHSM-hESM03 cell line is shown) is enhanced in naïve cells. Metaphase chromosomes were counterstained with DAPI (blue).

After transfer into naïve conditions and cultivation for 10 passages, three cell lines (H9, HUES9, and hESM01) retained their XCI characteristics including the absence of *XIST* expression (Figure 4A) and H3K27me3 foci, whereas the cell line hESM03 underwent significant changes in *XIST* and H3K27me3 foci expression (Figure 4A, 4B). However, XCI in hESM04 cells proved to be refractory to naïve conditions (Figure 4A).

Next, we decided to investigate whether epigenetic reprogramming to the naïve state can lead to reactivation of Xi in human PSCs thereby addressing the problem of XCI erosion [29]. Reactivation should be accompanied by an Xi replication time shift towards early replication; thus, we performed replication timing analysis in the NHSM-hESC lines. We considered replication late if EdU incorporation was observed within the pericentromeric autosome regions (in all primed ESCs except the primed HUES9 as shown in Figure 1E). Replication was considered early if EdU incorporation occurred in the euchromatic regions of autosomes and the active X chromosome (for example see the primed HUES9 cell line shown in Figure 1E).

In naïve conditions, the Xi acquired new bands of early replication in the middle part of the p-arm and in the middle of the q-arm in two cell lines, NHSM-hESM03 and NHSM-H9 (Figure 4C, Supplementary Figure 2A). These regions replicated synchronously with the active X chromosome.

Xi in primed HUES9 cells replicated synchronously with the active X except for only two bands. Transfer to naïve conditions did not change this pattern. We did not observe any additional bands of early replication (Supplementary Figure 2A).

The Xi in NHSM-H9 and NHSM-hESM03 cells resembled the pattern of replication in both the primed and NHSM-HUES9 cell lines. The distal part of the q-arm, the pericentromeric region and the distal part of the p-arm of Xi in the NHSM-H9 and NHSM-hESM03 cell lines retained its late replication (Figure 4C, Supplementary Figure 2A). The naïve NHSM-hESM01 cell line showed considerable cellular heterogeneity in the pattern of Xi replication (Supplementary Figure 2A). Synchronous replication was detected in the pericentromeric region of the q-arm or the distal part of the q-arm. A band in the middle of the p-arm retained synchronous replication in all NHSM-hESM01 cells.

Overall, we did not find any cells where two X chromosomes in the same cell exhibited identical patterns of EdU incorporation during the S phase. Thus, we concluded that transfer to naïve conditions led to a shift of replication, however, it did not fully reactivate the previously inactive X chromosome in NHSM-hESCs.

### 5-hydroxymethylcytosine reveals variability in reactivated regions

In previous studies, 5-hydroxymethylcytosine (5-hmC) was shown to be an important epigenetic modification associated with the pluripotent state [32] and excluded from the pericentromeric heterochromatin. Its distribution was correlated with the H3K4me1 and H3K4me2 chromatin modifications, and 5-hmC enrichment was found to be a marker of active X chromosome regions [33]. We performed 5-hmC immunostaining of metaphase chromosomes in NHSM-hESCs. In some cells, we detected the Xi with enrichment of 5-hmC within the same approximate regions that escaped from late replication (Figure 4D). However, the NHSM-hESCs in our study showed variable 5-hmC distribution between the cells and between the lines (Supplementary Figure 2B).

### Absence of H3K27me3 foci upon naïve hESC differentiation

**Figure 5:**
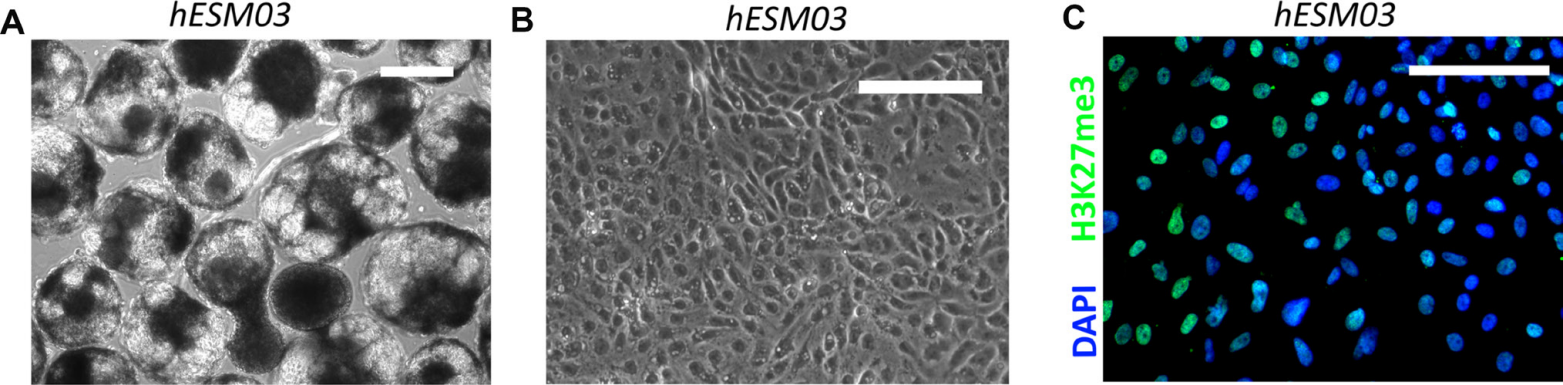
Reactivated Xi acquires an irreversible state during reprogramming in NHSM (**A**) Embryoid bodies (6 days after plating in low-adhesion dish) formed from the NHSM-hESM03 cell line. Phase contrast. Scale bar, 100 μM. (**B**) Differentiated cells. Phase contrast image of fibroblast-like cells differentiated from NHSM-hESM03 cells. (**C**) NHSM hESM03-derived differentiated cells stained with H3K27me3 (green) antibodies. H3K27me3 foci are not detected. Nuclei were counterstained with DAPI (blue). Scale bar, 100 μM.

In naïve culture conditions, the NHSM-hESC lines lost H3K27me3 foci and shifted their replication timing, thus demonstrating possible X chromosome reactivation. We chose the cell line NHSM-hESM03, which showed prominent changes in reactivation (loss of *XIST*, loss of H3K27me3 foci, and replication shift) during its transition towards the naïve state. The NHSM-hESM03 cell line was differentiated via the formation of the embryoid bodies (Figure 5A), and the cells were plated on a gelatin-coated dish for further spontaneous differentiation. After 14 days, the cells were examined for H3K27me3 by immunostaining. We did not find any prominent H3K27me3 foci in the differentiated cells (Figure 5B, 5C). Thus, we did not observe classical XCI in the differentiated NHSM-hESM03 cell line. This indicated that reactivation of the X chromosome in hESCs under NHSM conditions occurred in an aberrant manner.

## DISCUSSION

The heterogeneity of XCI status might hamper the application of human female PSCs for cell therapy, since the epigenetic instability of the inactivated X chromosome in female cells can lead to diseases including oncological and autoimmune [34, 35]. The role of stable epigenetic regulation is crucial for correct PSC differentiation [36]. hESCs are considered to exist in a primed pluripotent state because of their resemblance to mouse primed EpiSCs derived from post-implantation epiblast cells [4]. In our study, all five hESC lines demonstrated uniform morphological changes under naïve culture conditions, although only four cell lines were effectively passed through single-cell replating. It should be noted that there are no generally accepted criteria for the naïve pluripotent state. Thus, we evaluated expression changes in naïve-associated genes. Among the cell lines that were effectively replated by trypsinization, only two showed molecular signs of the naïve state and upregulated naïve state-associated genes (table summarizing data on X chromosome state in primed and NHSM conditions is shown in Supplementary Figure 3). This indicated that despite reported effectiveness [15, 26] the NHSM conditions were not able to overcome the intrinsic epigenetic heterogeneity of some cell lines for a uniform transition to the naïve state. However, all cell lines demonstrated similar morphological changes and we cannot rule out the possibility that extended passaging for an additional couple of dozens passages will overcome primed state.

Surprisingly, in two cell lines, we observed SSEA-4-positive and SSEA-4-negative colonies after as few as 5 passages in NHSM. A recent study revealed the same phenomenon: cultivation in 5i/L/A naïve conditions led to the emergence of SSEA-4-“negative” cells [31]. Moreover, the authors found that SSEA-4-“negative” cells seemed to exist in a more naïve state according to methylation data and their comparison with human epiblasts *in vivo*. Still, the authors used alternative naïve-cultivation conditions supplemented with LIF and Activin as well as inhibitors of the following kinases and pathways: ERK1/2, GSK3β, ROCK, BRAF, and SRC (5iLAF) [18]. Obviously, there is a close inter-relationship between the pluripotent marker SSEA-4 and the naïve state. Thus, loss of SSEA-4 presumably may become a precise marker of the naïve state, as well as CD24. The latter was previously shown to be negative in naïve [37].

The current view is that the developmental distance between the naïve and primed states lies in the XCI status difference between mouse and human ESCs [29]. In our study, only two cell lines (hESM03 and H9) significantly changed their XCI states. Along with the absence of H3K27me3 modification and *XIST* expression, these two cells lines acquired a shift in the replication timing of similar chromosome regions upon naïve culture conditions. The detected replication timing shift was towards the early S-phase of the cell cycle, which indicated that partial X chromosome reactivation had occurred. Moreover, these two cell lines were exactly the same lines that had previously been demonstrated by upregulation of naïve-associated genes to persist in a more naïve state. Thus, we considered that the naïve conditions used significantly affected the epigenetic state and X inactivation. Although functional reactivation still did not occur, the loss of inactive X markers and partial reactivation of X was irreversible, and upon differentiation from the “naïve” state, no conventional XCI occurred. Our results regarding the absence of “classical” XCI after differentiation are consistent with the data reported recently in which hESCs do not change their XCI status during differentiation [38].

It is interesting to note that the two X chromosome regions that displayed replication timing shifts were similar to those that reactivated after reprogramming from a somatic to a pluripotent state [28] and were also the same regions had increased transcriptional activity in a meta-analysis of iPSCs and ESCs [39]. In a recent study, reactivation of the X chromosome as a result of the erosion process in primed ESCs during prolonged cultivation was restricted to H3K27me3 domains [9]. The authors showed that loss of H3K27me3 was accompanied by loss of *XIST*, leaving the H3K9me3 regions unaffected. These X chromosome domains in the primed ESCs corresponded to the approximate regions of X reactivation we found in the hESM03 and H9 cells, but only after prolonged cultivation in NHSM conditions, suggesting that these domains are more epigenetically vulnerable.

In summary, our results demonstrated cell line-specific responses to the transition into naïve NHSM conditions during similar passaging conditions. We concluded that the NHSM naïve culture conditions epigenetically alter XCI status towards partial reactivation, although variability in XCI status in female human ESCs remains. Additionally, we observed some specific X chromosome regions that preserved their metastable states independently of growth conditions, thus suggesting some specific mechanisms of XCI.

However, we could not rule out the possibility that extended passaging of unresponsive cell lines in NHSM or alternative culture conditions would be able to overcome the X chromosome inactivation state in primed hESC lines. It was recently reported that X chromosome reactivation and biallelically expressed X-linked genes were detected in the H9 transgenic cell line under two alternative naïve culture conditions (5iL/A/F and 2i/L+PKCi) [23, 24]. Additionally, biallelic X-linked genes expression has been shown in the UCLA1 cell line under 5iL/A/F, although the level of the X-linked expression was similar to the level for the cells with monoallelic Xi [22]. Importantly, despite the fact that priming to the naïve resetting in 5iL/A/F conditions induced X chromosome reactivation, it was followed by non-random XCI upon differentiation [22, 38].

The reason for the inability to fully reactivate Xi in hESCs, as observed in mESCs, is an open question. It is likely that hESCs are incapable of existing in the absence of dosage compensation. In human cells at the blastocyst stage, biallelic expression is detected in epiblasts, although this expression is already compensated for according to the dosage at this stage [40]. The same phenomenon was reported under 5i/L/A-naïve hESC conditions. In that study, along with the reactivation of the previously inactive X, chromosome-wide transcriptional dampening was detected [22], confirming the hypothesis that human cells *in vivo*, starting from the ICM stage, and *in vitro* are unable to exist with a double dose of X-linked genes. Thus, *in vitro* attempts to obtain cells with two fully reactivated X chromosomes result in two possible scenarios: either X chromosome transcriptional dampening occurs or monoallelic expression of conventionally primed ESCs persists with partial reactivation of regions for which biallelic expression is not crucial.

## MATERIALS AND METHODS

### Cell culture

The hESC lines, hESM03, hESM04 and hESM01, have been previously described [41–43]. The HUES 9 cell line was a generous gift from Prof. D. Melton [44]. The H9 cell line was provided by the WiCell Research Institute, Inc., Madison, Wisconsin.

The primed ESC lines were maintained in 20% O_2_ on a feeder layer of Mitomycin C-inactivated mouse embryonic fibroblasts (MEFs) in ES medium containing DMEM/FF-12 medium (Paneco) with 20% KO serum replacement (Invitrogen), 2 mM L-glutamine, 50 units/mL penicillin, 50 μg/mL streptomycin, 0.1 mM β-mercaptoethanol, 1% nonessential amino acids (all from Paneco) and 4 ng/ml bFGF (Peprotech). The cells were manually replated every 4-6 days.

The naïve hESC lines were cultivated according to a previously published protocol [15]. The NHSM-hESC lines were grown in 5% O_2_ in DMEM/FF-12 medium containing 20% serum replacement, 2 mM L-glutamine, 50 units/mL penicillin, 50 μg/mL streptomycin, 0.1 mM β-mercaptoethanol, 1% nonessential amino acids and 4 ng/ml bFGF and the following small molecule inhibitors: 1 mkM PD0325901 (Stemgent), 3 mkM CHIR99021 (Tocris), 10 mkM SP600125 (Tocris), and 10 mkM SB203580 (Stemgent). Y-27632 (ROCKi, 5 mkM) (Stemgent) was used 24 h after replating in some cell lines. The cells were passaged every 5 days. During passaging, the cell colonies were treated with 0.05% trypsin-EDTA (HyClone), and a single-cell suspension was then prepared and transferred to new Petri dishes.

For differentiation, NHSM-hESCs were treated with 0.05% trypsin, and 2 × 10^6^ cells were placed on AggreWell plates (Stem Cell Technologies) in ES media. The following day, the formed embryoid bodies were collected and placed in an ultralow adhesion culture well (Corning). The medium was changed every 2 days and contained 15% FBS (HyClone), 2 mM L-glutamine, 50 units/mL penicillin, 50 μg/mL streptomycin, and 1% nonessential amino acids. After 14 days of differentiation, the embryoid bodies were dissociated and replated on gelatin-covered plates for further analysis.

### Metaphase spread preparation

For preparation of metaphase spreads, colcemid (Invitrogen) was added 30 min before harvesting at a final concentration of 0.1 μg/mL. The cells were trypsinized (0.05% trypsin, HyClone) for 1–2 min at room temperature, the trypsin was inactivated with FBS (HyClone), and the cells were incubated in hypotonic buffer (10 mM Tris-HCl; 10 mM NaCl; and 5 mM MgCl_2,_ pH 7.5) for 15 min at 42° C. The cell suspension was then treated with a methanol-acetic acid fixative, according to a standard protocol modified as described previously [45]. The metaphase spreads were used for immunostaining with anti-5hmC antibody and for detection of incorporated EdU by click chemistry.

### Pulse-labeling using EdU

Cells were pulse-labeled for 20 min with EdU at a final concentration of 1 μM. Metaphase spreads were prepared 7 h after pulse-labeling using a standard cytogenetic protocol. Incorporated EdU was visualized by click reaction with fluorescent azide. The click reaction was performed on slides with metaphase spreads using the Click-It kit (Invitrogen) according to the manufacturer's instructions. At least 20 metaphase spreads were analyzed for each cell line.

### 5-hmC specific immunostaining of metaphase chromosomes

Slides with metaphase spreads were incubated in 4 N HCl for 15 min at room temperature. The slides were then incubated for 30 min at room temperature in blocking solution containing 2.5% BSA, 2.5% goat serum, and 0.1% Tween20 in PBS. The slides were incubated overnight at 4° C with a primary anti-5hmC antibody diluted in blocking solution. The primary antibody used was a polyclonal rabbit anti-5hmC (Active Motif, 1:1000). After 3 washes in PBS/0.1% Tween20, the metaphase spreads were stained with Alexa Fluor 488-conjugated goat anti-rabbit IgG (Invitrogen, 1:1000) for 1 h at room temperature. At least 15 metaphase spreads were analyzed for each cell line.

### DNA-FISH

X chromosomes were identified using fluorescent *in situ* hybridization with the SE X-centromeric probe for X chromosome DXZ1 (Kreatech) according to the manufacturer's instructions, performed after a click reaction or 5-hmC immunostaining.

### Immunohistochemistry

Cells were grown on dishes and fixed with 4% paraformaldehyde/PBS for 10 min at room temperature and washed three times with PBS. Fixed cells were permeabilized in PBS/0.5% Triton X100 for 10 min, blocked with blocking solution (2% BSA, 2% goat serum, and 0.1% Tween20 in PBS) and incubated with a primary antibody diluted in blocking solution overnight at 4° C. The cells were then washed three times with PBS/0.01% Tween20, incubated with secondary antibodies for 1 h at room temperature, washed in PBS/0.05% Tween20, counterstained with DAPI, mounted with Vectashield (Vector Laboratories) and imaged. The following primary antibodies were used: rabbit polyclonal H3K27me3 diluted 1:5000 (Abcam), mouse SSEA-4 1:10 (DSHB), rabbit polyclonal OCT4 1:100 (Abcam), and mouse monoclonal TRA-1-60 1:500 (Abcam). The secondary antibodies used were as follows: goat anti-rabbit Alexa Fluor 488-conjugated goat IgG (Invitrogen, 1:1000) and goat anti-mouse Alexa Fluor 555-conjugated goat IgG (Invitrogen, 1:1000).

### RNA isolation and cDNA synthesis

RNA was isolated using the QIAGEN RNA isolation kit (RNeasy Mini Kit). Two micrograms of RNA was used for the reverse transcription reaction with the Promega M-MLV RT Kit. Twenty-five microliters of the reverse transcription mix was diluted 40 times, and 2 μl of this mix was used as a template for PCR. Quantitative real-time PCR with the intercalating dye SYBR Green was performed in triplicate on a 7500 Applied Biosystems and IQ-5 BioRad thermal cycler. The experiment was repeated 3 times. The RT-PCR primers used were as follows:

GAPDH-forward: TGTTGCCATCAATGACCCC TT; GAPDH-reverse: CTCCACGACGTACTCAGCG; TBX3-forward CTTACCAGCCACCATCCACC; TBX3- reverse: GATCAGTTTCACAAGCGGGG; KLF4-forward: CCCACATGAAGCGACTTCCC; KLF4-reverse: CAGGTCCAGGAGATCGTTGAA; NANOG-forward: TTTGTGGGCCTGAAGAAAACT; NANOG-reverse: AGGGCTGTCCTGAATAAGCAG.

## SUPPLEMENTARY MATERIALS AND FIGURES



## Abbreviations

bFGF: basic fibroblast growth factor
EpiSC: epiblast stem cells
ESC: embryonic stem cells
ICM: inner cell mass
LIF: leukemia inhibitory factor
NHSM: naïve human stem cell medium
PCR: polymerase chain reaction
PSC: pluripotent stem cells
RT-PCR: real-time PCR
SSEA-4: stage-specific embryonic antigen-4
XCI: X-chromosome inactivation
Xi: inactive X chromosome
