# Supplementary material for: Epigenetic reprogramming by naïve conditions establishes an irreversible state of partial X chromosome reactivation in female stem cells
Source: Oncotarget. 2018 May 18;9(38):25136–47. doi: 10.18632/oncotarget.25353 (PMC5982739; doi:10.18632/oncotarget.25353)
Supplement: Supplementary file 1 [file oncotarget-09-25136-s001.pdf]

Epigenetic reprogramming by naïve conditions establishes an irreversible state of partial X chromosome reactivation in female stem cells

SUPPLEMENTARY MATERIALS

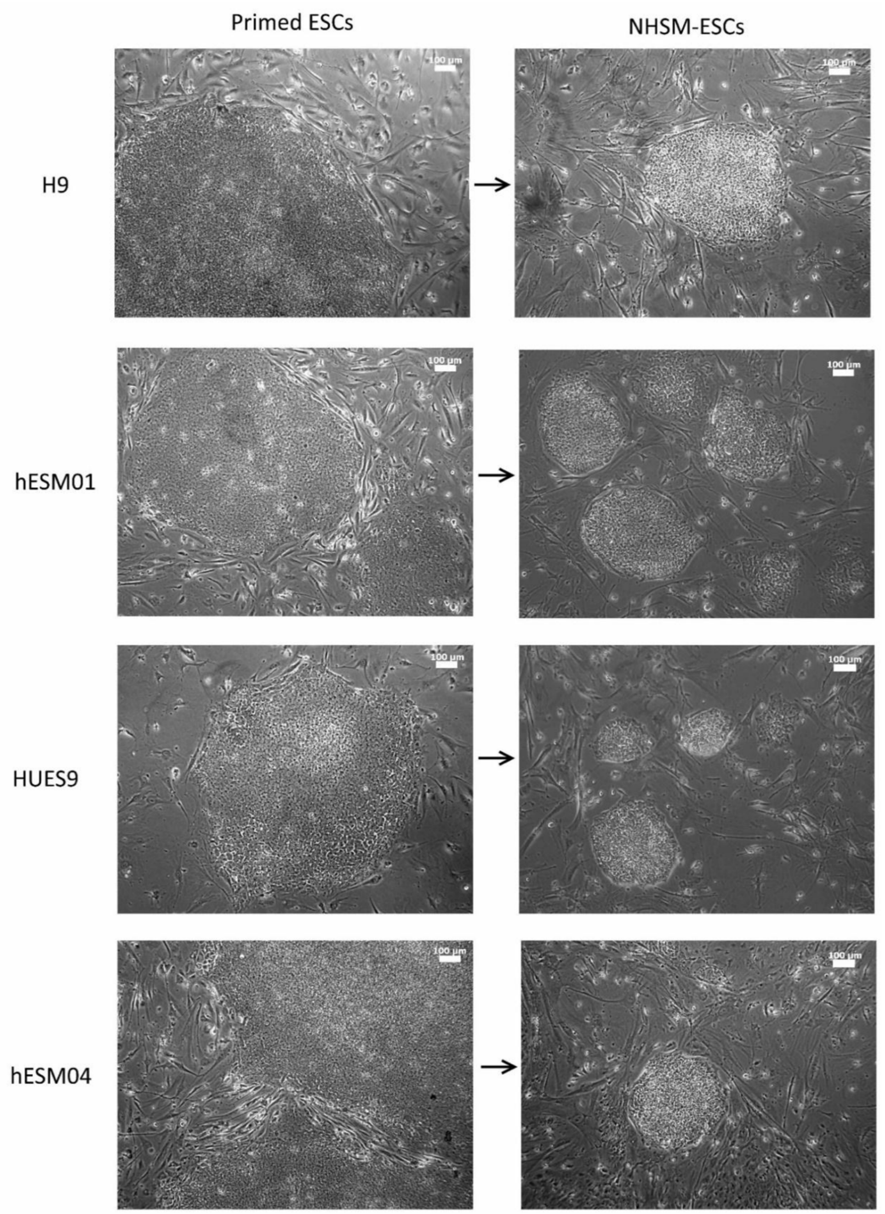

Supplementary Figure 1: hESC colonies' morphology before and after cultivation in NHSM for 10 passages.

A

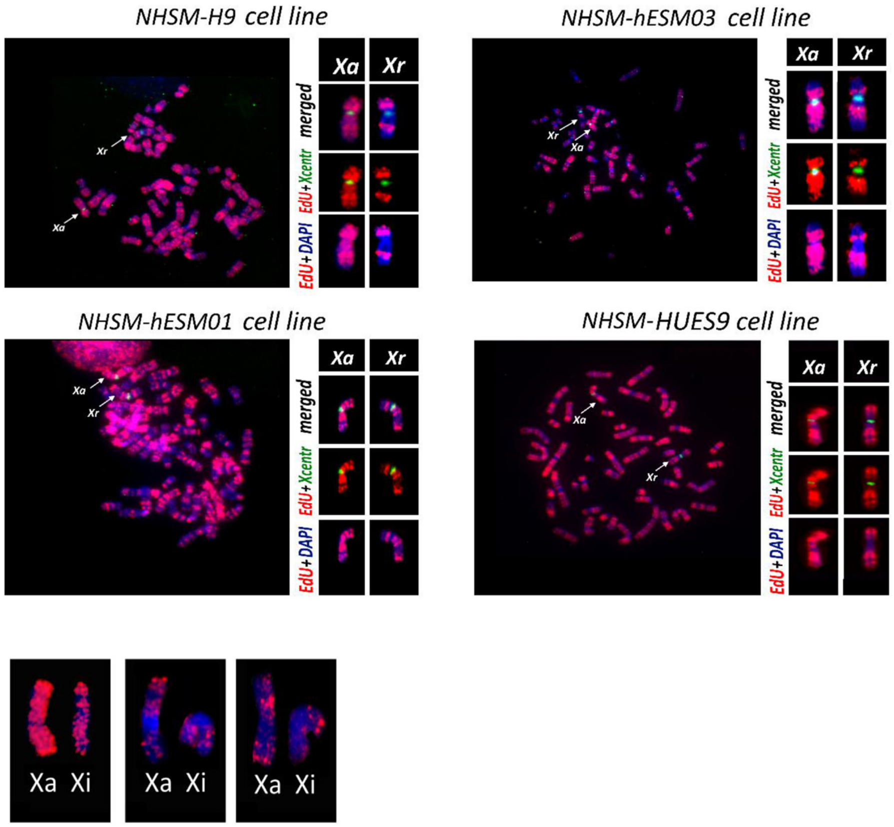

B

Supplementary Figure 2: Replication timing analysis and 5-hmC distribution of X chromosomes in NHSM-hESC lines. (**A**) Bands of synchronous replication are shown in red. NHSM-H9 and NHSM-hESM03 cells lines demonstrated alike pattern of replication: distal band of p-arm, distal region of q-arm and pericentomeric region retain asynchronous replication. HUES9 cell line kept distal region of q-arm and pericentomeric region in asynchronous replication. hESM01 cell line showed variability in regions of synchronous replication. (**B**) Comparison of 5-hmC distribution between Xi chromosomes for hESM03 cell line after 10 passages of cultivation in naïve conditions. Xa – active X chromosome, Xi – inactive X chromosome, Xr – partly reactivated X chromosome.

	H3K27me3		XIST		Synchronous replication of the second X	
	Primed	NHSM	Primed	NHSM	Primed	NHSM
HUES9	−	−	−	−	Some bands	Some bands
hESM01	−	−	+ weak	−	no	Different bands in different cells
H9	−	−	−	−	no	Some bands
hESM03	+	−	+	−	no	Some bands
hESM04	+	+	+	+	no	no

Supplementary Figure 3: Table summarizing data on XIST expression, H3K27me3 foci and replication timing in 5 primed hESC lines before and after cultivation in NHSM conditions for 10 passages.
